# Supplementary material for: Advanced Spectroscopic Studies of the AIE-Enhanced ESIPT Effect in a Selected 1,3,4-Thiadiazole Derivative in Liposomal Systems with DPPC
Source: Int J Mol Sci. 2025 Oct 31;26(21):10643. doi: 10.3390/ijms262110643 (PMC12607980; doi:10.3390/ijms262110643)
Supplement: Supplementary file 1 [file ijms-26-10643-s001.zip › ijms-3937738-supplementary.pdf]

International Journal of *Molecular Sciences*

MDPI

Article

Advanced Spectroscopic Studies of the AIE-Enhanced ESIPT Effect in a Selected 1,3,4-Thiadiazole Derivative in Liposomal Systems with DPPC

Alicja Skrzypek [1], Iwona Budziak-Wieczorek [1,*], Lidia Ślusarczyk [2], Andrzej Górecki [3], Daniel Kamiński [4], Anita Kwaśniewska [5], Sylwia Okoń [6], Igor Różyło [7] and Arkadiusz Matwijczuk [2,*]

1 Department of Chemistry, Faculty of Food Sciences and Biotechnology, University of Life Sciences in Lublin, Akademicka 15, 20-950 Lublin, Poland; alicja.skrzypek@up.lublin.pl
2 Department of Biophysics, Faculty of Environmental Biology, University of Life Sciences in Lublin, Akademicka 13, 20-950 Lublin, Poland; lidia.slusarczyk@up.lublin.pl
3 Department of Physical Biochemistry, Faculty of Biochemistry, Biophysics and Biotechnology, Jagiellonian University, Gronostajowa 7, 30-387 Kraków, Poland; andrzej.gorecki@uj.edu.pl
4 Department of Chemistry, Maria Curie-Sklodowska University, Akademicka 19, 20-033 Lublin, Poland; daniel.kaminski@mail.umcs.pl
5 Department of Applied Physics, Faculty of Mechanical Engineering, Lublin University of Technology, Nadbystrzycka 38, 20-618 Lublin, Poland; a.kwasniewska@pollub.pl
6 Institute of Plant Genetics, Breeding and Biotechnology, University of Life Sciences in Lublin, 20-950 Lublin, Poland; sylwia.okon@up.lublin.pl
7 Faculty of Medicine, Medical University of Lodz, Al. Kościuszki 4, 90-419 Łódź, Poland; rozyloigor@gmail.com
* Correspondence: iwona.budziak@up.lublin.pl (I.B.-W.); arkadiusz.matwijczuk@up.lublin.pl (A.M.)

Academic Editor: Bruno Rizzuti

Received: 2 October 2025
Revised: 29 October 2025
Accepted: 30 October 2025
Published: 31 October 2025

Citation: Skrzypek, A.; Budziak-Wieczorek, I.; Ślusarczyk, L.; Górecki, A.; Kamiński, D.; Kwaśniewska, A.; Okoń, S.; Różyło, I.; Matwijczuk, A. Advanced Spectroscopic Studies of the AIE-Enhanced ESIPT Effect in a Selected 1,3,4-Thiadiazole Derivative in Liposomal Systems with DPPC. *Int. J. Mol. Sci.* **2025**, *26*, 10643. https://doi.org/10.3390/ijms262110643

Copyright: © 2025 by the authors. Licensee MDPI, Basel, Switzerland. This article is an open access article distributed under the terms and conditions of the Creative Commons Attribution (CC BY) license (https://creativecommons.org/licenses/by/4.0/).

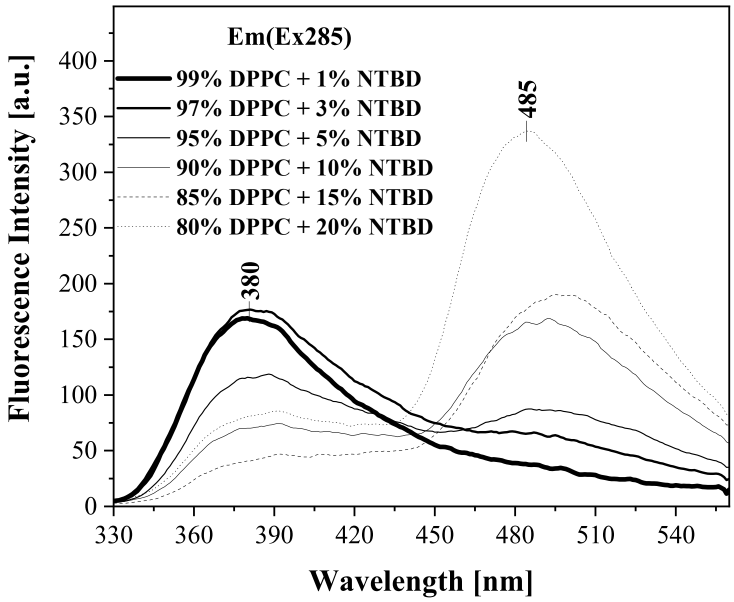

Figure S1. Electronic emission spectra of NTBD in the DPPC liposomal system at 285 nm excitation wavelength obtained at room temperature.

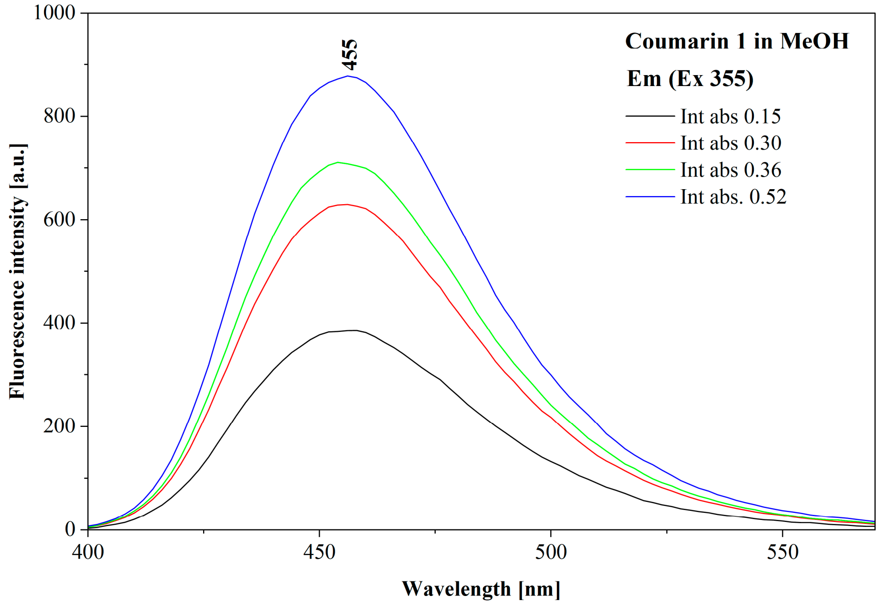

Figure S2. Electronic emission spectra of coumarin1 in methanol at 355 nm excitation wavelength obtained at room temperature. The intensity of the absorption maximum is given in the legend.

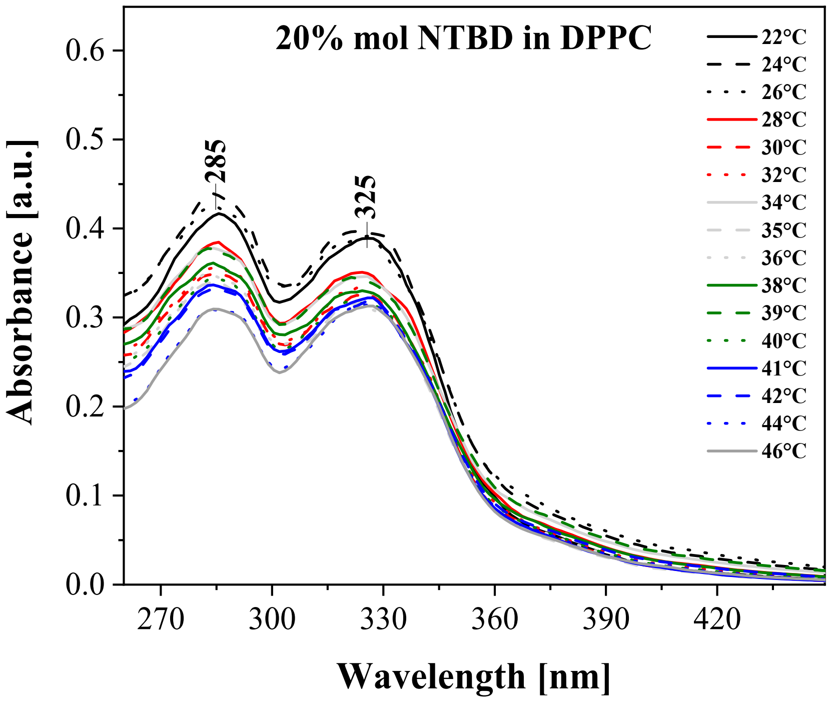

Figure S3. Electronic absorption spectra for 20% mol NTBD in DPPC liposomal system obtained at different temperatures.

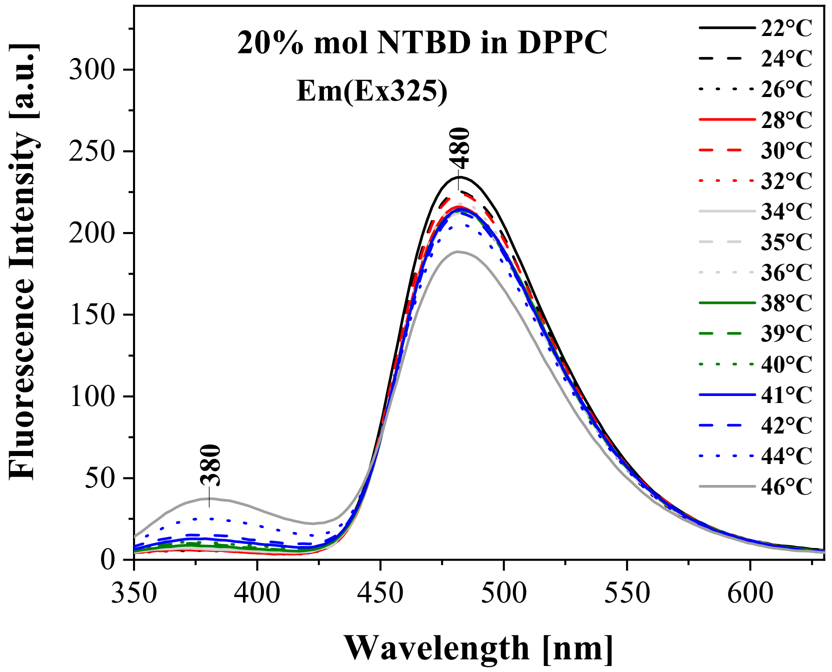

Figure S4. Electronic emission spectra for 20% mol NTBD in DPPC liposomal system obtained at different temperatures, 325 nm was chosen as the excitation wavelength.

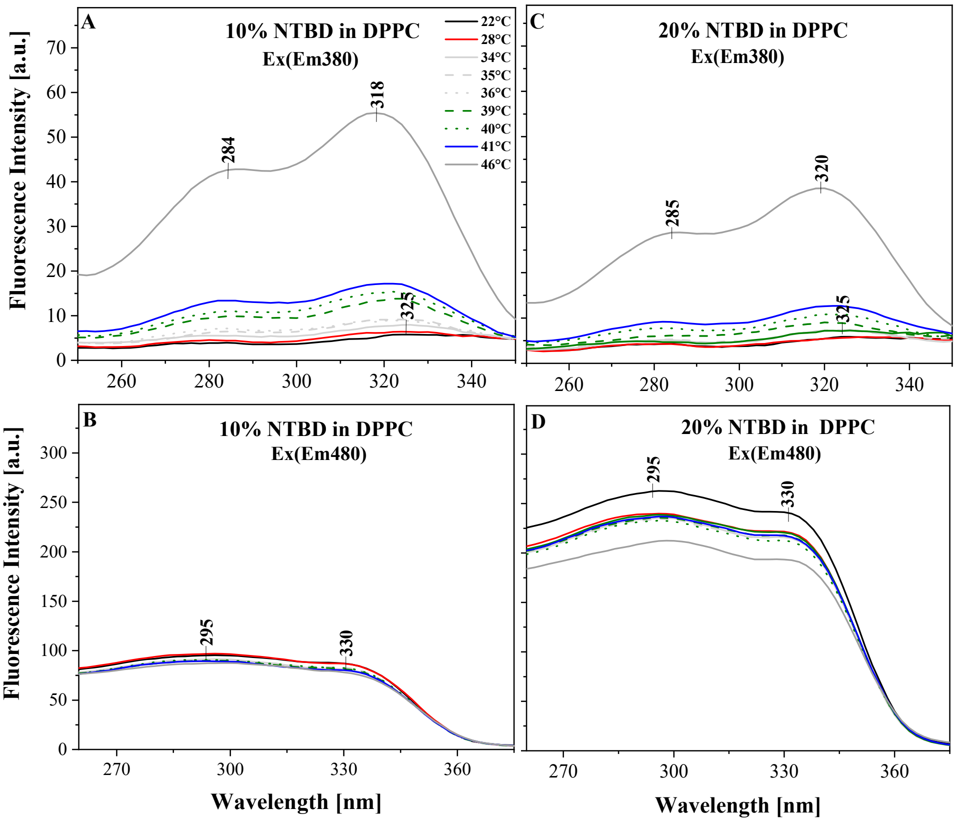

Figure S5. Excitation spectra for NTBD in DPPC as a function of temperature. Panels A and B 10% mol NTBD. Panels C and D 20% mol NTBD. Excitation wavelength 380 nm in panels A and C and 480 nm in panels B and D.

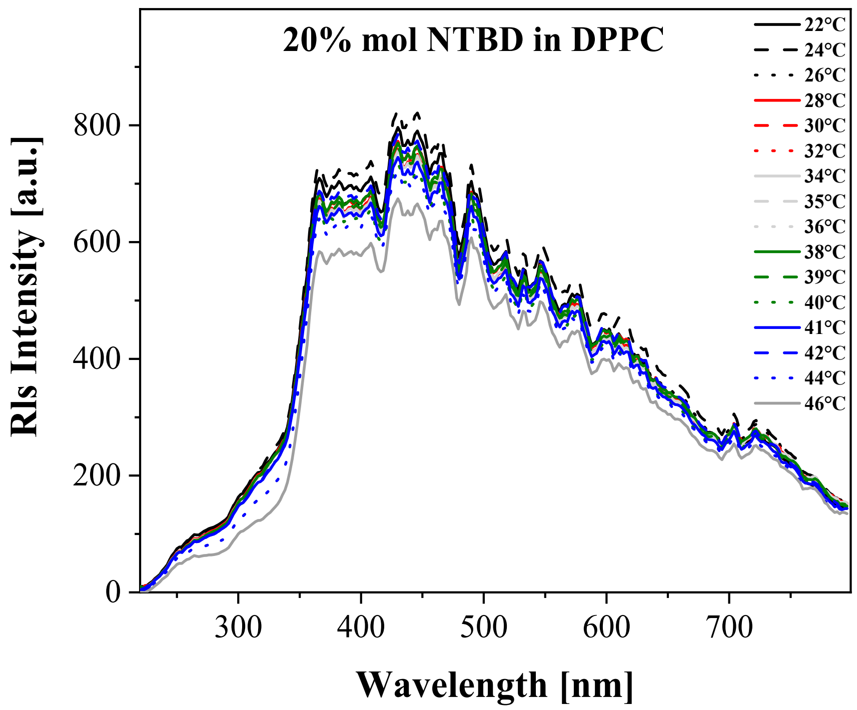

Figure S6. Resonant light excitation spectra RLS for NTBD 20% mol in DPPC liposomal system obtained at different temperatures.

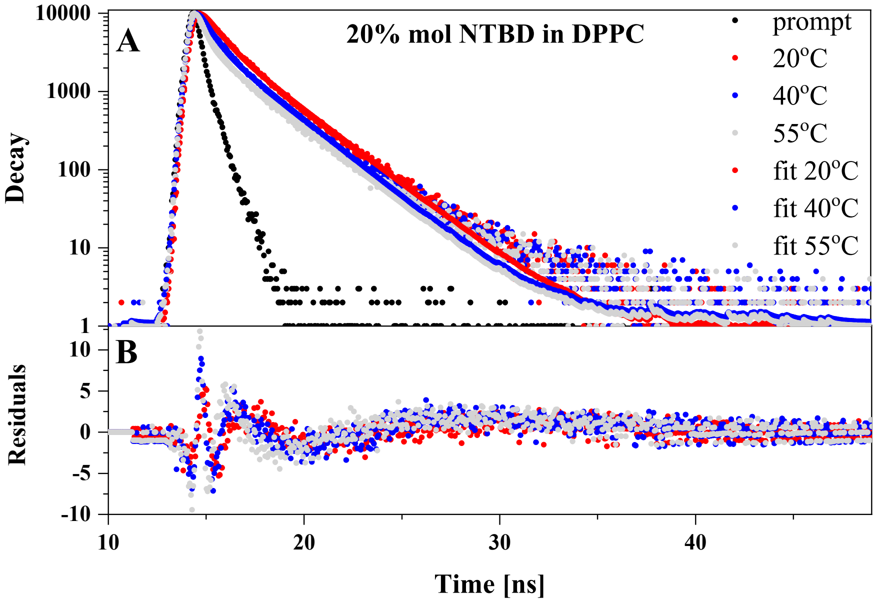

Figure S7. Panel A: Typical fluorescence decay profiles obtained for liposomal system with NTBD at 20% mol concentrations at various temperatures 20 ^{0}C, 40 ^{0}C and 55 ^{0}C. The decays are presented as points, and their respective 2-exponential fits as solid lines. Panel B: residual distributions.

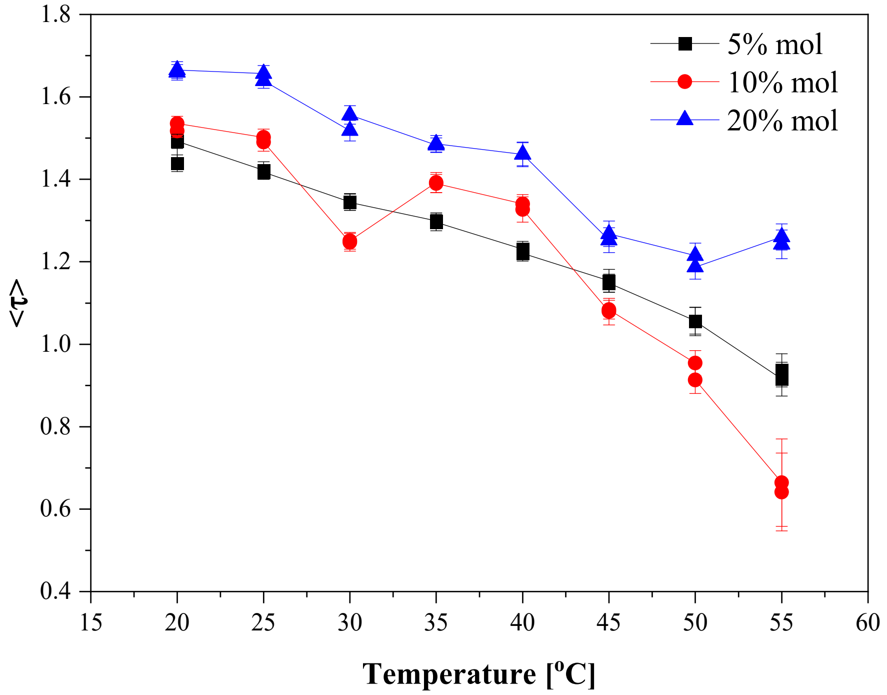

Figure S8. Mean fluorescence lifetime <τ> with standard deviation for NTBD at different concentrations in the DPPC liposomal system with increasing temperature.

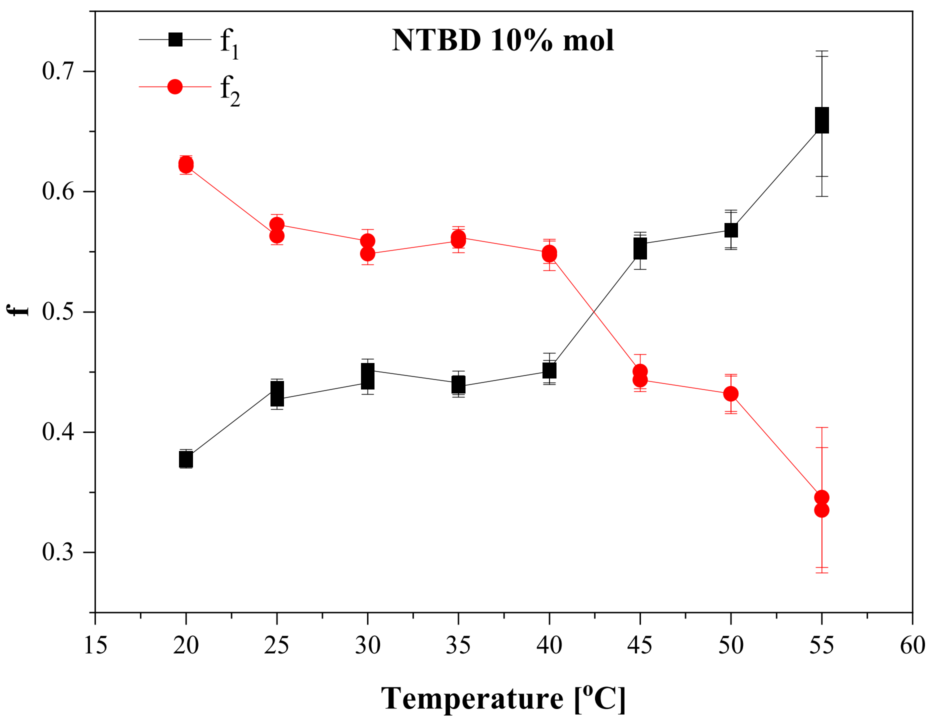

Figure S9. Intensity of f1 and f2 contributions with standard deviation for 10% mol NTBD in the DPPC liposomal system with increasing temperature.

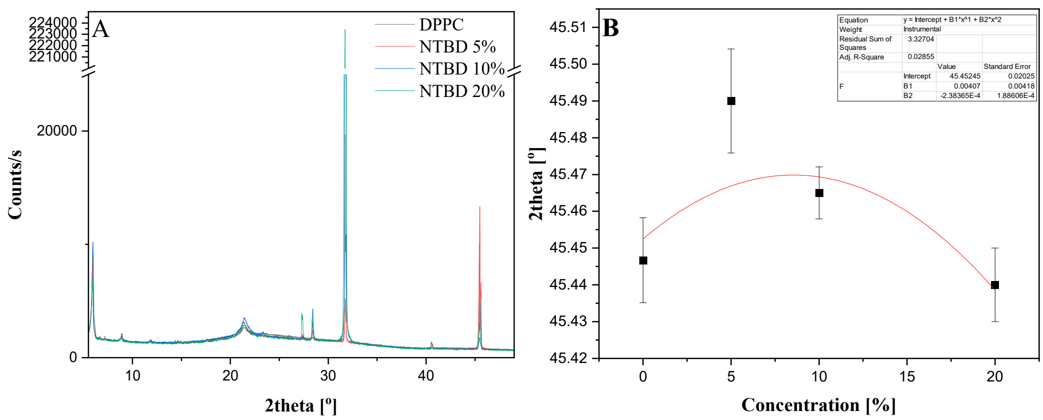

Figure S10. The X-ray diffraction data from multibilayers of DPPC containing NTBD compound at concentrations of 5%, 10% and 20% mol DPPC-Panel A, Panel B- dependence of the 2theta [°] with the standard deviation depending on the concentration of the NTBD compound.

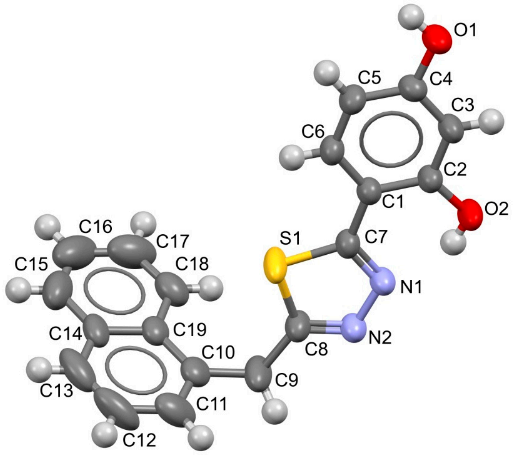

Figure S11. Structure of the NTBD molecule with atom numbering and thermal ellipsoids (50% probability), derived from X-ray diffraction data.

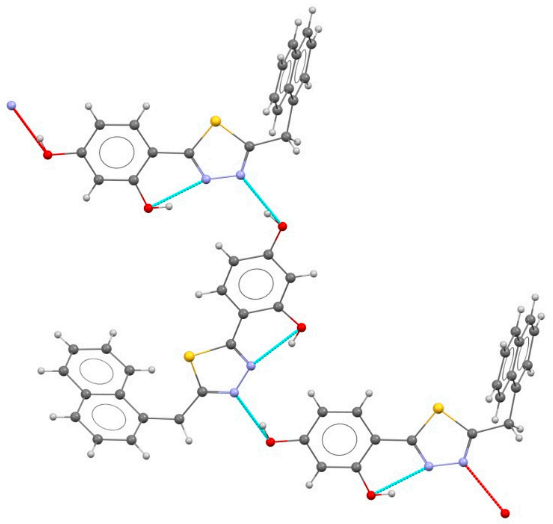

Figure S12. Hydrogen bonds between NTBT molecules in crystal net.

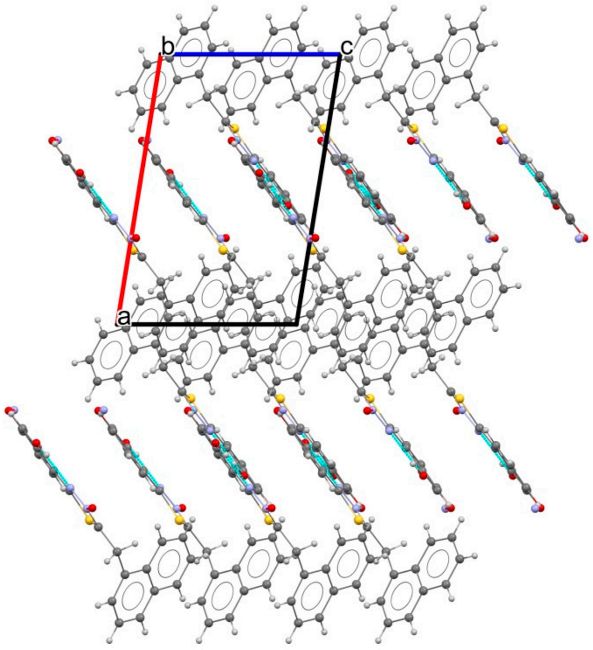

Figure S13. Depiction of the alternating arrangement of hydrophilic layers (resorcyl and thiadiazole rings) and hydrophobic layers (naphthalene and methylene groups) in the NTBD molecule.

Table S1. Polydispersity index (PDI) values obtained from DLS analysis.

Sample	PDI
DPPC	0.593
DPPC + 1% NTBD	0.496
DPPC + 3% NTBD	0.557
DPPC + 5% NTBD	0.202
DPPC + 10% NTBD	0.502
DPPC + 15% NTBD	0.681
DPPC + 20% NTBD	0.596
